# Hypoglossal nerve palsy in infectious mononucleosis and *Fusobacterium necrophorum* tonsillitis: a case report and literature review

**DOI:** 10.1186/s13052-025-01905-z

**Published:** 2025-10-06

**Authors:** Simone Scannapiecoro, Giuseppe Indolfi, Vincenzo Temperino, Sandra Trapani

**Affiliations:** 1 Department of Health Sciences, University of Florence, Florence, Italy; 2 Department NEUROFARBA, University of Florence, Florence, Italy; 3 Meyer Children’s Hospital IRCCS, Viale Pieraccini 24, Florence, 50139 Italy

**Keywords:** Hypoglossal nerve palsy, Infectious mononucleosis, Fusobacterium necrophorum tonsillitis, Case report

## Abstract

**Background:**

Hypoglossal nerve palsy (HNP), although rare, can result from various causes and more commonly in children is secondary to infectious etiologies. Its optimal management requires accurate diagnosis and exclusion of severe complications like Lemierre’s syndrome, vascular events, tumors, or demyelination/inflammatory processes).

**Case presentation:**

A previously healthy 16-year-old boy was diagnosed with HNP, infectious mononucleosis and *Fusobacterium necrophorum* tonsillitis. The patient presented with fever, neck swelling, and swallowing difficulty, accompanied by left-side tongue deviation. Prompt treatment with clindamycin and prednisone led to rapid improvement of symptoms (within 5 days) and complete recovery after 4 weeks.

**Conclusions:**

This case highlights the importance of considering uncommon neurological complications in patients with common diseases. An extensive literature review on HNP in childhood was conducted, summarizing the diverse etiologies, clinical presentations, diagnostic tests, and treatment approaches.

## Background

Hypoglossal nerve palsy (HNP) is an uncommon neurological abnormality presenting with atrophy of the tongue musculature and tongue deviation [1]. This condition, although rare, can occur in various clinical contexts. The most frequent cause of paresis is nerve compression due to the presence of tumors or head injury in adults [2], whereas, in children, several infectious etiologies should be considered, such as infectious mononucleosis and *Fusobacterium necrophorum* pharyngotonsillitis [3]. Understanding its etiology and clinical implications is crucial for appropriate management and treatment decisions. This case report describes the unusual presentation of HNP in a 16-year-old boy diagnosed with infectious mononucleosis and *Fusobacterium necrophorum* pharyngotonsillitis. We report the clinical features, diagnostic approach, and management strategies, highlighting the importance of considering rare neurological complications in the setting of common infectious diseases. Furthermore, we thoroughly examined the literature on HNP in childhood and concisely summarized the various etiologies, clinical manifestations, and approaches for diagnosis and treatment.

## Case presentation

**Fig. 1:**
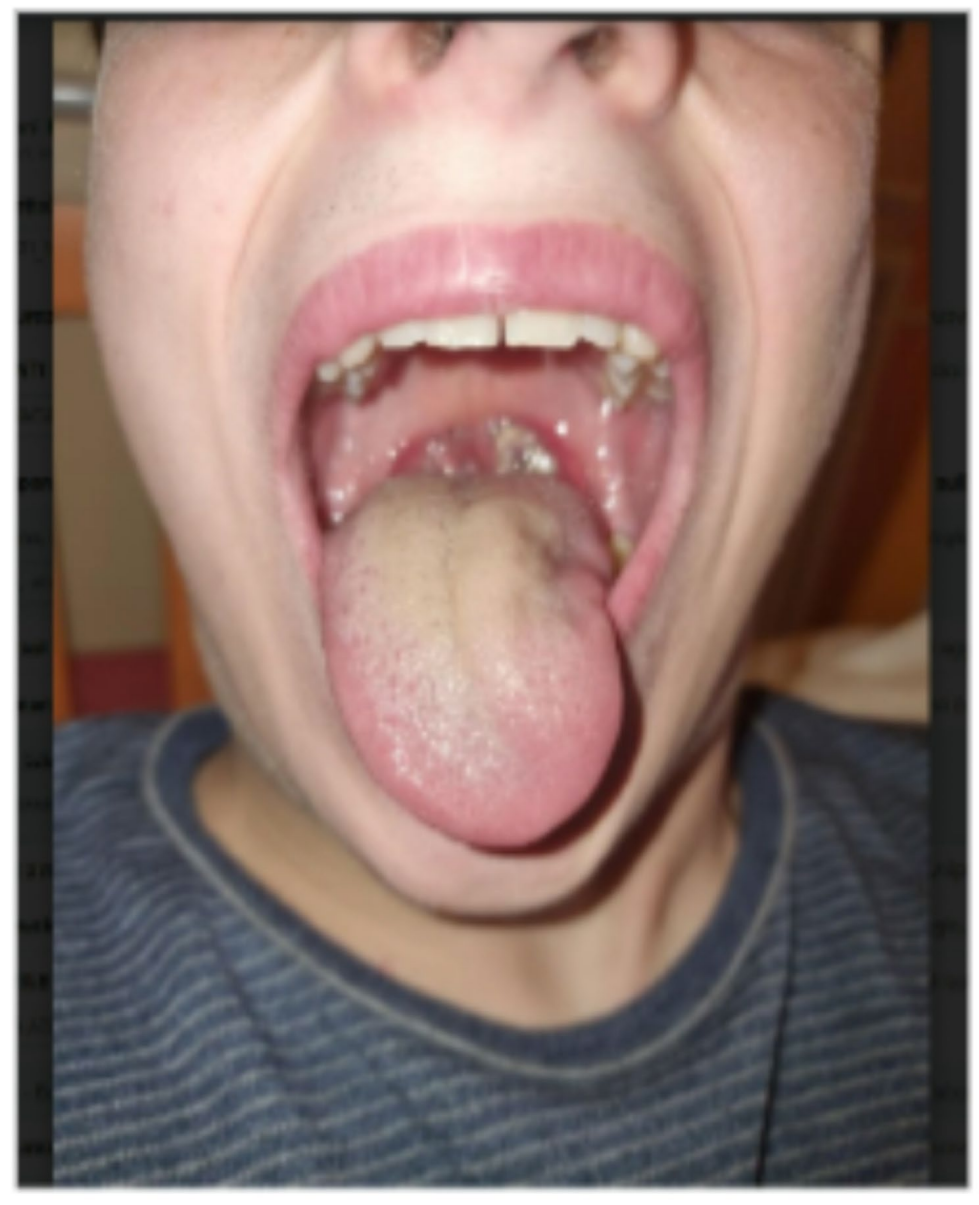
Left XII nerve palsy: the tongue deviates to the left when protruded

**Fig. 2:**
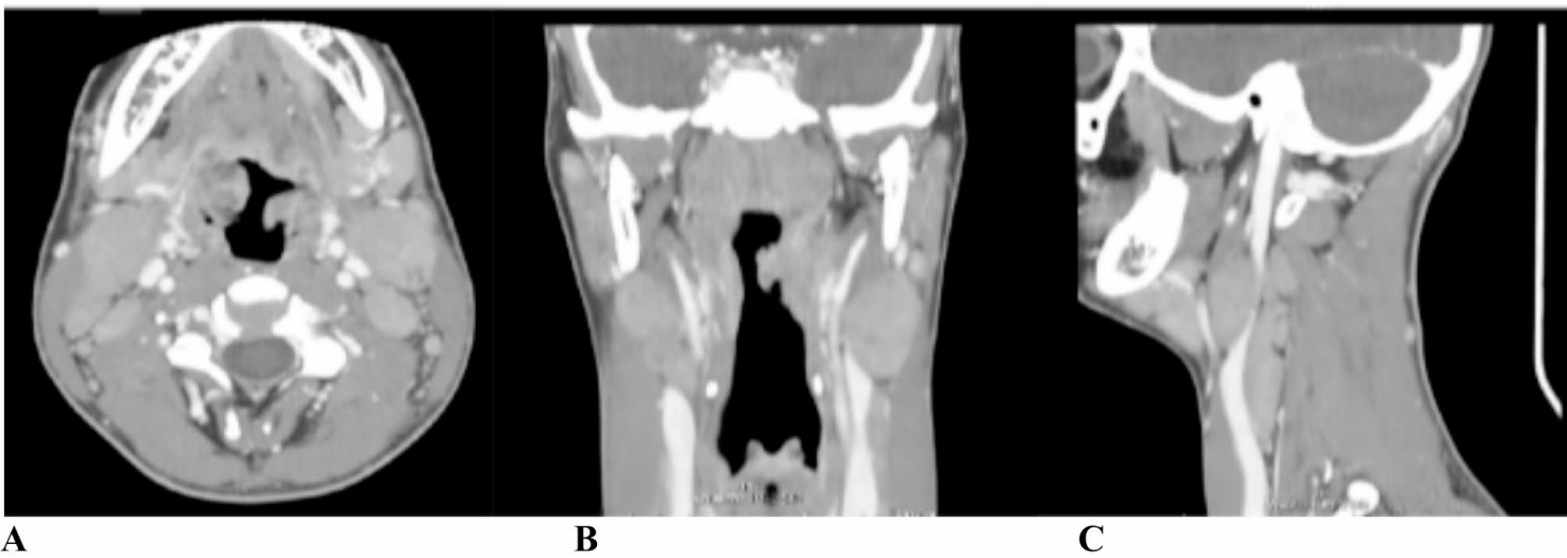
**A**: Significant enlargement of the palatine tonsils (especially on the right side) showing heterogeneous density due to liquefactive aspects. **B/C**: Multiple coarse cervical lymphadenopathies more evident at levels II, III, and IV bilaterally; these are lymph nodes with a hyperplastic-reactive appearance without signs of incipient liquefaction

A previously healthy 16-year-old boy presented fever, neck swelling, and pharyngodynia for 1 week. **His medical history included a hospitalization at 6 months of age for bronchiolitis and a previous injury involving a distraction of the anterior superior iliac spine 6 years ago. Family history revealed non-consanguineous parents**,** with the father deceased due to pancreatic cancer**,** a maternal aunt affected by type 1 diabetes mellitus**,** and two healthy 19-year-old heterozygous twin sisters. No other significant familial conditions were reported**. Blood tests showed leukocytosis 17,400 cell/mmc (17% neutrophils, 66% lymphocytes, 16.4% monocytes), elevated C-reactive protein (CRP, 2.6 mg/dL), elevated liver enzyme levels (aspartate aminotransferase -AST- 167 UI/L, alanine aminotransferase -ALT- 210 UI/L), and lactate dehydrogenase (LDH, 592 U/L). Epstein-Barr Virus (EBV) serology was positive for viral capsid antigen IgM but negative for anti-Epstein-Barr nuclear antigen (EBNA) IgG, confirming the acute infection. The child was started on oral ibuprofen (600 mg 4 times daily) and prednisone (25 mg twice daily) for 4 days. Subsequently, he started complaining of swallowing and feeding difficulties, was referred to the Emergency Department (ED) and then admitted to the Pediatric Unit. Physical evaluation on admission, revealed fever, rhinolalia, left lateral neck mass, torticollis, bilateral painful supraclavicular and latero-cervical lymphadenopathies. The oropharyngeal inspection showed occlusive tonsillar hypertrophy with white-grey exudate and left-sided tongue deviation without hemi-atrophy or fasciculations, consistent with peripheral hypoglossal nerve palsy (Fig. 1). Cranial nerve XI function was normal; there was no weakness in the trapezius or the sternomastoid muscle. Blood tests showed leukocytosis (32.510 cells/mmc with neutrophils 27.6%, lymphocytes 48.6%, monocytes 4.9%, **large unstained cells (luc) 15.9%**), AST 102 UI/L, ALT 340 UI/L, LDH 545 UI/L, and CRP 2.14 mg/dL; renal and liver function tests were normal. Real-time Polymerase Chain Reaction on a throat swab was positive for *Fusobacterium necrophorum*, and antibiotic therapy with clindamycin was started. The neck computed tomography (CT) scan was performed to evaluate HNP and to rule out complications such as Lemierre’s syndrome or pharyngeal or neck abscesses and showed areas of liquefaction in the tonsillar context, multiple coarse cervical lymphadenopathies are more evident at levels II, III, and IV bilaterally with a hyperplastic-reactive appearance without signs of incipient liquefaction (Fig. 2). During hospitalization, the patient was treated with clindamycin (600 mg 3 times per day) and prednisone (25 mg twice daily) for 5 days. Five days after admission, the HNP had significantly improved, along with other associated symptoms and inflammatory markers normalized. The HNP completely recovered in 4 weeks.

## Discussion and conclusions

**Our case report presents an unusual and original instance of hypoglossal nerve palsy (HNP) in a pediatric patient**,** resulting from the concurrent infections of *Fusobacterium necrophorum *and Epstein-Barr Virus (EBV). This dual etiology**,** rare even in adult cases**,** represents a significant addition to the pediatric HNP literature**,** where infectious causes predominate. The clinical complexity of this case highlights the importance of considering rare neurological complications in infectious diseases and stresses the role of early imaging to rule out severe conditions like Lemierre’s syndrome.**

The hypoglossal (12th ) nerve is responsible for the motor innervation of the tongue, allowing for chewing, swallowing, and speech. Anatomically, its nucleus is located at the posterior aspect of the medulla oblongata, it arises between the olive and pyramid as 10–15 rootlets. Its fibres exit the posterior cranial fossa through the hypoglossal canal just in front of the foramen magnum; then, the nerve descends into the neck beneath the sternocleidomastoid muscle, it courses between the small rectus muscle of the head and the internal carotid artery (ICA), medial to the glossopharyngeal, vagus, and accessory nerves. Continuing its path between the ICA and the jugular vein, this nerve reaches the stylian muscles, crosses the lateral surface of the external carotid artery, reaches the anterior margin of the sternocleidomastoid muscle, and enters the tongue. The pathway through the neck requires careful consideration due to its proximity to the anatomical structures mentioned earlier. This delicate route is susceptible to various conditions that elevate pressure, such as tumors, reactive or malignant cervical lymphadenopathy, vascular complications like Lemierre syndrome, or traumatic/iatrogenic causes; all these factors can potentially lead to paralysis [2].

Kane et al. reviewed a series of 100 adults with HNP and highlighted the different etiologies: neoplasia, which is the most common found in 49 cases, trauma (12 cases), stroke (six cases), hysteria (six cases), multiple sclerosis (six cases), surgical causes (five cases), Guillain-Barre syndrome (four cases), and infectious origin (four cases) [3]. To investigate the pathogenesis of HNP in childhood, we conducted an extensive literature review, carefully selecting all the pediatric cases (Table 1). To the best of our knowledge, 27 cases (including our boy) have been reported (17 males, 65%) with a mean age of 11.8 years (range < 1 to 17 years). Whereas HNP was secondary to different conditions in most children, it has been found to be idiopathic only in two cases [4–6]. Unlike adulthood where infectious diseases are uncommon (4%), in reported children an infectious origin was identified in most cases (16 cases, 59%) [7–8, 10–21]. Other causes include inflammatory conditions (2 cases of hypertrophic pachymeningitis) [22, 23], hematologic/oncologic diseases (2 cases) [24, 25], trauma (2 cases) [26, 27], iatrogenic (1 case) [28], neurovascular conflict (1 case) [29], and post-vaccination (1 case) [30].

**Table 1:** Cases of pediatric hypoglossal nerve palsy in literature: etiology, clinical presentation, complications, treatment, and prognosis

Author (ref)	Age/ sex	Etiology	Clinical presentation	Laboratory Tests	Radiology studies	Complications	Treatment (duration)	Prognosis
He J et al. [7]	17/M	*F. necrophorum*	Sore throat Vomit Sepsis Fever Hypotension Tachycardia Neck stiffness Purulent tonsils cervical LAP	WBC 20,000 cells/mmc CRP 25 mg/dL creatinine 1.7 mg/dL urea 14.5 mmol/L PLT 16,000 cells/mmc	CT scan: L-sided thrombus in the IJV, SS and CS; patchy consolidation in both upper lobes of the lungs; arthritis of the left atlanto-occipital and C1/C2 facet joints; small L lateral epidural abscess MRI head and c-spine: L VA and ICA thrombosis, skull base osteomyelitis and an extensive epidural abscess	Lemierre Syndrome	Ceftriaxone (nr) Meropenem (nr) Metronidazole (nr) Warfarin (nr)	Nr
Blessing K et al. [8]	12/M	*F. necrophorum* (PCR from the drainage specimen)	Sore throat Neck pain Fever Cervical LAP Torticollis L tongue deviation hypotension Desaturation	WBC normal PLT 100,000 cells/mmc CRP 20.6 mg/dL Creatinine 1.9 mg/dL AST 170 U/L ALT 125 U/L	MRI: inflammatory lesions dorsally and laterally to the L occipital condyle (suspected thrombophlebitis of the L JIV) Chest X-ray: bilateral pulmonary lesions CT scan: cervical LAP, torticollis of the atlanto-axial joint; fluid collection in the left mastoid air cells and middle ear; suspected retropharyngeal hypodensity	Lemierre Syndrome Mastoiditis	Cefuroxime (day 1) Meropenem (day 2–19) Clindamycin (day 2–19) Ampicillin/Sulbactam (day 20–26) Dopamine (nr) LD-heparin (3 months) Intubation CPAP (nr) Mastoidectomy (nr)	19 Days (almost complete recovery
Wrigh LKD et al. [10]	16/F	Mononucleosis	Sore throat Tonsillitis Cervical LAP R tongue deviation	ESR 25 mm/h WBC 5,900 cells/mmc EBV fluorescent antibody titre 1:256)	X-ray of skull and mastoid: normal	Absent	No therapy	24 Weeks
Sibert JR et al. [11]	17/M	Mononucleosis	Sore throat Headache Nausea Malaise Fever Cervical LAP Tonsillitis	WBC 11,900 cells/mmc (70% atypical monocytes) CSF: normal	No radiological test performed	Absent	Penicillin V (nr) Steroids (8 days)	6 Months (not full recovery)
Parano E et al. [12]	7/M	Mononucleosis	Sore throat Cervical LAP Mild splenomegaly R tongue deviation	WBC 19,700 cells/mmc Atypical lymphocytosis (40%) VCA IgG-M 1:100 EBV EA IgG 1:200 EBNA negative	CT scan of the skull base and upper cervical region: normal MRI of the same region: normal	Absent	No therapy	8 Days improved 21 Days full recovery
Carra-Dallière C et al. [13]	16/M	Mononucleosis	Dysarthria Fever Sore throat Cervical LAP Malaise Anorexia Weight loss R tongue deviation, atrophy, hypoesthesia		Brain MRI: normal ENG: negative Facial EMG: normal CSF: normal	Absent	No therapy	2 Months
van Baalen A et al. [14]	9/F	Mononucleosis	L tongue deviation	Nr	MRI: normal CSF: normal	Nr	Nr	Nr
Johns MM et al. [15]	18/F	Mononucleosis	Sore throat “Muffled” voice Dysphagia Cervical LAP Pharyngitis	40% mononuclear cells 13% atypical lymphocytes	Videostroboscopy: high-grade left vocal fold paresis CT scan of skull base and neck: normal	Absent	Steroids (3 days)	7 Months full recovery
Maddern BR et al. [16]	15/M	Mononucleosis	Severe tonsillitis Hoarseness R tongue deviation	Monospot test: positive	Videostroboscopy: right vocal cord immobile	Absent	Cefotaxime (nr) Steroids (nr) Adeno- tonsillectomy	6 Months
Derrick J [17]	9/F	Mononucleosis	Fever, Coryza Sore neck Sore throat Cervical LAP Halitosis R tongue deviation Hepatomegaly	CRP 7.3 mg/dL WBC 2,220 cells/mmc EBV IgM positive EBNA IgG negative	Abdomen US: normal MRI head: normal	Absent	Steroids (nr)	Not improved at 2 months
Zafeiriou DI [18]	6/M	Mononucleosis	Low grade fever Sore throat Malaise Dysarthria L tongue deviation	Nr	Nr	Nr	Nr	Nr
Gunawardana SS et al. [19]	11/M	Tuberculosis (M. tuberculosis from drainage)	Dysphagia Weight loss Sore throat L neck swelling Neck stiffness L tongue deviation	Nr	MRI neck/skull: extensive cystic/solid mass in the retropharyngeal and prevertebral spaces, with extension to the base of skull	Nr	Antitubercular therapy	Nr
Chatterjee S et al. [20]	Nr	Tuberculosis	Craniovertebral tuberculosis	Nr	Nr	Nr	Antitubercular therapy	Nr
Hadjikoutis S [21]	14/F	Streptococcal Pharyngitis	Tongue heaviness R tongue deviation and hemiatrophy	ASO > 1280	MRI brain: R hemiatrophy of the tongue with fatty change	Absent	Nr	3 Months partially improved
Stricker T et al. [22]	7/F	Suppurative lymphadenopathy	Dysarthria Odynophagia Tonsillitis Cervical LAP L tongue deviation	WBC 7800 cells/mmc ESR 44 mm/h CRP 2.6 mg/dL ASO: 200–400 UI/mL VCA IgG > 1:640 IgM negative	CT scan head/neck: nodular 2 × 2 × 1.5 cm lesion; contrast-enhancing in the left retro-pharynx at C2 Chest X-ray: normal	Absent	Ceftriaxone IV (7 days)	2 Months Almost full recovery 4 Months full recovery
Yoon JH et al. [5]	11/M	Idiopathic	Dysarthria R tongue weakness	WBC 9,300 cells/mmc PLT 382,000 cells/mm	MRI brain: normal CT scan neck: normal	Absent	Steroids (12 days)	15 Days
Sugama S et al. [6]	8/M	Idiopathic	Dysarthria L tongue deviation	Nr	X-ray skull: normal CT scan brain: normal	Nr	Multivitamins (nr)	12 Weeks
Hsieh DT et al. [23]	16/M	Hypertrophic Pachymeningitis	headache R tongue deviations	CRP, ANCA, ACE, CSF analysis, QFT test: normal/negative Biopsy: negative	MRI brain: extensive Gd-enhancing pachymeningeal thickening involving the posterior convexities and posterior fossa Chest CT scan: normal	Absent	Steroids (nr) Rituximab (2 doses)	Not improved at 2 years
Dziedzic T et al. [24]	18/M	Hypertrophic Pachymeningitis after AOM	Painful edema on the L side tongue Dysphagia Hoarseness L XII nerve palsy L soft palate paresis	Nr	MRI brain: hypointense lesion in T1 and T2 with contrast enhancement of posterior fossa and hypoglossal canal. CT scan: no widening of the hypoglossal canal on the L side compared to the R; no osteoblastic or osteoclastic changes around the canal.	Absent	Partial condylectomy	Nr
Woo E et al. [25]	12/M	Metastatic germinoma (after ventriculoatrial shunt)	Dysarthria R hypoglossal palsy	βHCG: 2650 mUI/mL CSF analysis: normal	CT scan: resolution of the suprasellar mass, no tentorial enhancement or bony erosion. Occlusion of the R IJV, enlargement of the vessel, enhancing luminal filling defect. Cerebral angiography: vascular mass (4 × 2 cm) at the neck R side supplied by branches of the R ECA. Partial occlusion of the R TS and SSs up to the R IJV	Nr	Radiotherapy Cisplatin (nr) VP16 (nr)	Nr
Cantalupo G et al. [26]	16/M	Diffuse large cell lymphoma (Tapia’s syndrome)	Transitory loss of consciousness L lymph nodes abscess L tongue deviation ORL: hypomobility of left hemilarynx	Nr	ECG, TTE, TCD, EEG, chest CT-scan: normal MRI: enlarged lymph nodes in the L jugular-digastric, retro-para-pharyngeal spaces and posterior triangle, widespread infiltration of the neurovascular from C5 to the jugular foramen Biopsy (jugular lymph nodes): high-grade peripheral B-cell lymphoma (diffuse large cell lymphoma).	Absent	Nr	Nr
Brennan RJ et al. [27]	11/M	Trauma (head injury)	Severe dysarthria Headache Neck pain Trysma	Nr	Skull cervical spine, chest, abdomen, pelvis, hips X-ray: normal Cerebral CT-scan: normal	Nr	Nr	3 Days
Kwon TH et al. [28]	11/F	Trauma (retroclival epidural hematoma)	GCS 15 Headache Neck pain Dysarthria Bilateral abducens nerve palsy L uvula deviation Weakened tongue’s movement	Nr	Brain CT-scan: hyperdense lesion ventral to the medulla C-spine X-ray: straightening of the C-spine lordosis, large prevertebral swelling in front of the clivus and upper C-spine C-spine CT-scan: acute retroclival EDH from dens to the upper clival region (the dura and tectorial membrane had been stripped off the clivus by the hematoma)	Nr	C-spine immobilization with brace	6 Weeks
Ginsburg GM et al. [29]	12/M	Iatrogenic (Halo-suspension traction)	Difficult swallowing Decreased intake Inability to protrude	Nr	CT-scan and MRI: normal	Absent	Decreased weight of Halo-suspension Steroids (nr)	6 Weeks full recovery
Toldo I et al. [30]	3/F	Neurovascular Conflict	R tongue atrophy fasciculation partial deficit of motility Mild sialorrhea Rhinolalia	Nr	MRA brain: elongated and tortuous VAs with neuro-vascular compression of the medulla oblongata at the R corticospinal tract and R inferior olivary nucleus with involvement of the interposed olivary sulcus. CT scan: atlo-occipital malformation, hypoglossal foramen stenosis EMG and SEPs: normal	Absent	Nr	Nr
Felix JK et al. [31]	< 1/M	Post influenza vaccination	Fever R tongue deviation Purulent rhinorrhea	Nasopharyngeal culture positive for S. pneumoniae	Nr	Nr	Penicillin V (nr)	12 Weeks (full recovery)
*Scannapiecoro S et al.*	16/M	Mononucleosis *F necrophorum*	Fever Neck swelling Pharyngodynia Rhinolalia L torticollis LAP (cervical and supraclavicular) Occlusive and essudative tonsillar hypertrophy L tongue deviation	WBC 32.510 cells/mmc Monocytes 4.9% LUC 15.9% AST 102 UI/L ALT 340 UI/L LDH 545 UI/L CRP 2.14 mg/dL F. necrophorum on throat swab (*PCR)*	CT-scan neck: areas of liquefaction in the tonsillar context	Absent	Clindamycin (2 weeks) Steroids (2 weeks)	4 Weeks

Legend: this table summarizes reported cases of hypoglossal nerve palsy in pediatric patients, including details on the patient’s age and sex, the etiology of the condition, clinical presentation, laboratory test results, radiological studies conducted, complications, treatment administered, and final prognosis. Each row of the table corresponds to a documented case, with references to the original authors and publications. Where information is absent or not reported, it is indicated as “NR” (Not Reported) or “absent.”

List of Abbreviations

Nr: not reported; LAP: lymphadenopathy; L: left; R: right, CRP: C reactive protein, IJV: internal jugular vein, WBC: white blood cells, PLT: platelets, CT-scan: computed tomography scan, MRI: Magnetic Resonance Imaging, US: ultrasonography, ICA: internal carotid artery, PCR: polymerase chain reaction, AST: aspartate aminotransferase, ALT: alanine aminotransferase, LDH: lactate dehydrogenase, CPAP: Continuous Positive Airway Pressure, ESR: erythrocyte sedimentation rate, EBV: Epstein Barr Virus, CSF: cerebrospinal fluid, EMG: electromyography, ENG: electroneurography, TTE: transthoracic echocardiography, ASO: antistreptolysin O, ANCA: antineutrophil cytoplasmic antibodies, ACE: angiotensin converting enzyme, QFT: quantiferon test, OAM: acute otitis media, MRA: magnetic resonance angiography, Gd: gadolinium, VA: vertebral artery, ECA: external carotid artery, CS: cavernous sinus, TS: transverse sinus, SS: sigmoid sinus SEPs: somatosensory evoked potentials, Anti-VCA: viral capsid antigen, βHCG: free beta-subunit of human chorionic gonadotropin, ORL: othorinolaringoiatry, GCS: glasgow coma scale, EDH: epidural hematoma LUC: large unstainded cells

In the infectious subgroup, the most common infection was infectious mononucleosis. HNP related to infectious mononucleosis was reported in nine cases [9–17, 31]. In these cases, the palsy is more commonly reversible, unilateral, and isolated [3]; multiple cranial nerve palsy was described in only two cases [14–15]. EBV infection is associated with a wide range of acute neurologic diseases in children including encephalitis, meningitis, mononeuropathies, optic neuritis, transverse myelitis, cerebellar ataxia, peripheral neuropathy, and cranial nerve palsy with facial nerve being the most often affected [31]. Two cases of HNP were described in association with Lemierre’s syndrome, a possibly life-threatening complication of *Fusobacterium necrophorum* pharyngotonsillitis, which tends to have a more severe course [7–8]. In the case described in the present report, given the coexistence of infectious mononucleosis and *Fusobacterium necrophorum* tonsillitis, Lemierre syndrome was ruled out by the CT scan. Tuberculosis has been described as the cause of HNP in two children, causing a retropharyngeal abscess in one [18] and cervical tuberculosis with condylar involvement in the other [19].

In nearly all the described children with HNP, the most common clinical manifestations were fever, sore throat, cervical lymphadenopathy, and neck pain/swelling/stiffness with torticollis. Halitosis, although typically present in tonsillitis due to *Fusobacterium necrophorum*, was not observed in the reported cases, besides our boy.

**The detection of Fusobacterium necrophorum in our patient’s throat swab was based on PCR testing**,** as no culture was performed. Despite the absence of a culture**,** the use of PCR is considered a reliable diagnostic tool**,** with studies demonstrating that it performs comparably to culture in identifying Fusobacterium necrophorum. According to Klug et al.‘s meta-analysis**,** recovery rates of Fusobacterium necrophorum using PCR and culture in patients with acute tonsillitis are statistically similar**,** reinforcing PCR’s validity as an alternative to culture. Considering the clinical presentation of our patient and the radiological findings (peri/tonsillar abscess)**,** which are more consistent with active infection rather than colonization**,** the role of Fusobacterium necrophorum as a co-contributive pathogen is strongly supported. This aligns with the observed clinical features**,** including fever**,** tonsillar exudates**,** and cervical lymphadenopathy. These characteristics suggest that Fusobacterium necrophorum**,** alongside the primary Epstein-Barr Virus (EBV) infection**,** likely played a role for the patient’s condition** [32].

Radiological investigations, such as neck CT and or MRI, performed in almost all cases were crucial to rule out Lemierre’s syndrome and detect other underlying pathologies, such as vascular events, tumors, or demyelination/inflammatory processes. Regarding therapy, isolated HNP does not require any specific treatment and the appropriate approach should be targeted on the etiology of each case, often necessitating antibiotic treatment depending on the identified pathogen when the palsy is related to bacterial infection. Corticosteroid therapy has been frequently used regardless of the specific etiology. Rare exceptions, such as cases due to trauma, neoplastic conditions, or iatrogenic injury (Table 1), can require a definite medical or surgical approach. With regards to prognosis, the recovery period, documented in 12 cases, had a median duration of 12 weeks.

HNP should be suspected when tongue deviation or hemiatrophy, fasciculations, or dysarthria are present, often associated with hyporexia/weight loss. In these cases, it is important to evaluate the coexistence of paralysis of other cranial nerves and perform a complete neurological evaluation. The isolated HNP is an uncommon finding in both infectious mononucleosis and *Fusobacterium necrophorum* pharyngotonsillitis, especially in children. The significance of our case highlights the need to consider rare complications of common conditions. When tonsillitis is associated with lymphadenopathy, it is crucial to perform a comprehensive neurological examination, paying special attention to evaluating the cranial nerves, including the 12th. If there is a neurological involvement, as HNP in our case, it is mandatory to conduct advanced imaging such as CT or MRI to exclude Lemierre’s syndrome and all possible causes of increased pressure in the cervical region. Conducting a comprehensive diagnostic workup is crucial for determining the appropriate approach.

## Data Availability

Not applicable.

## Abbreviations

ALT: Alanine aminotransferase
AST: Aspartate aminotransferase
CRP: C-reactive protein
CT: Computed tomography
EBNA: Epstein-Barr nuclear antigen
EBV: Epstein-Barr Virus
ED: Emergency Department
HNP: Hypoglossal nerve palsy
ICA: Internal carotid artery
LDH: Lactate dehydrogenase
LUC: Large unstained cells
MRI: Magnetic resonance imaging
